# Prolonged cetuximab treatment promotes p27^Kip1^-mediated G1 arrest and autophagy in head and neck squamous cell carcinoma

**DOI:** 10.1038/s41598-021-84877-4

**Published:** 2021-03-04

**Authors:** Kohei Okuyama, Keiji Suzuki, Tomofumi Naruse, Hiroki Tsuchihashi, Souichi Yanamoto, Atsushi Kaida, Masahiko Miura, Masahiro Umeda, Shunichi Yamashita

**Affiliations:** 1 Department of Oral and Maxillofacial Surgery, Graduate School of Medical and Dental Sciences, Tokyo Medical and Dental University, 1-5-45, Yushima, Bunkyo-ku, Tokyo, 113-8510 Japan; 2 Department of Radiation Medical Sciences, Atomic Bomb Disease Institute, Nagasaki University, Nagasaki, Japan; 3 Department of Clinical Oral Oncology, Nagasaki University Graduate School of Biomedical Sciences, Nagasaki University, Nagasaki, Japan; 4 Department of Maxillofacial Diagnostic and Surgical Science, Field of Oral and Maxillofacial Rehabilitation, Graduate School of Medical and Dental Sciences, Kagoshima University, Kagoshima, Japan; 5 Department of Cancer Biology, The University of Kansas Medical Center, Kansas City, KS USA; 6 Division of Oral Health Science, Department of Oral Radiation Oncology, Graduate School of Medical and Dental Sciences, Tokyo Medical and Dental University, Tokyo, Japan; 7 Center for Global Exchange, Fukushima Medical University, Fukushima, Japan; 8 Center for Advanced Radiation Emergency Medicine, National Institutes for Quantum and Radiological Science and Technology, Chiba, Japan

**Keywords:** Cancer, Cell biology, Molecular biology, Oncology

## Abstract

Cetuximab, an anti-epidermal growth factor receptor (EGFR) monoclonal antibody, is an efficient anti-tumor therapeutic agent that inhibits the activation of EGFR; however, data related to the cellular effects of prolonged cetuximab treatment are limited. In this study, the long-term cellular outcome of prolonged cetuximab treatment and the related molecular mechanism were explored in a head and neck squamous cell carcinoma cell line constitutively expressing a fluorescent ubiquitination-based cell cycle indicator. Fluorescent time-lapse imaging was used to assess clonal growth, cell motility, and cell-cycle progression. Western blot analysis was performed to measure the level of phosphorylation and protein-expression following cetuximab treatment. Over 5 days cetuximab treatment decreased cell motility and enhanced G1 phase cell arrest in the central region of the colonies. Significantly decreased phosphorylation of retinoblastoma, Skp2, and Akt-mTOR proteins, accumulation of p27^Kip1^, and induction of type II LC3B were observed over 8 days cetuximab treatment. Results of the present study elucidate the cetuximab-dependent inhibition of cell migration, resulting in high cell density-related stress and persistent cell-cycle arrest at G1 phase culminating in autophagy. These findings provide novel molecular insights related to the anti-tumor effects of prolonged cetuximab treatment with the potential to improve future therapeutic strategy.

## Introduction

Squamous cell carcinoma (SCC) is the most common type malignant tumor which develops in head and neck region. Despite advances in recent decades in diagnosis and improvement of imaging modalities, the survival of head and neck SCC (HNSCC) patients has remained unchanged^1–3^. This is due to the high recurrence rate and the high risk of cervical lymph node or distant metastasis^1–3^. The current standard treatment for HNSCC in most patients is surgery, and postoperative concurrent chemoradiotherapy using platinum-based agents is a widely accepted standard of treatment for the patients with the high risk of recurrence as determined by surgical pathological findings^4–6^. On the other hands, the therapeutic strategies for patients with locoregional recurrent or distant metastatic HNSCC are limited, and such patients have a median survival of 6 months and an expected 1-year survival rate of only 20%^7^.

Cetuximab is a chimeric IgG1 monoclonal antibody that binds to the extracellular domain of the epidermal growth factor receptor (EGFR) with high affinity^8^. Cetuximab blocks EGFR activation by preventing tyrosine kinase-mediated EGFR phosphorylation^8,9^. EGFR overexpression has been frequently observed in HNSCC and is thought to correlate with carcinogenesis, metastasis, the clinical stage, and a poor prognosis^10–12^. Systemic chemotherapy for HNSCC is associated with significant toxicity, which highlights the need for more targeted therapeutics^13^. The clinical efficacy of cetuximab was demonstrated in the landmark EXTREME study, which showed improved survival compared with a conventional standard chemotherapy regimen for recurrent or metastatic HNSCC^14^. Furthermore, promising results were obtained by administering cetuximab in combination with platinum-based agents in such cases, or as a radiosensitizer as a part of definitive radiotherapy for medically unfit patients who were unable to receive platinum-based agents^14,15^. The use of cetuximab has become a standard therapeutic regimen in the treatment of HNSCC^16–18^. The National Comprehensive Cancer Network Clinical Practice Guidelines in Oncology also recommended the inclusion of cetuximab in the systemic therapy regimen for advanced cases of HNSCC^19^. Based on the above findings, systemic therapy with cetuximab was selected for treating unresectable, recurrent, or metastatic HNSCC and had yielded favorable outcomes^20^.

In addition to the inhibition of EGFR activation, the anti-tumor effect of cetuximab is mediated by antibody-dependent cell-mediated cytotoxicity^12,21,22^, hypoxic tumor microenvironment induced drug resistance^23^, and inhibition of epithelial–mesenchymal transition^24^. Ohnishi et al. reported that environmental stimuli alter the activation of proteins associated with EGFR signal transduction, resulting in a change in cetuximab sensitivity^25^. Moreover, their results also indicated that cetuximab treatment markedly inhibited the migratory activity of tongue cancer cells; however, the underlying mechanism was unclear^25^.

Because EGFR inhibition by cetuximab can inhibit intracellular-signaling pathways linked to dysregulated cell growth, the anti-tumor effects of cetuximab are expected to involve cell cycle arrest. Indeed, cetuximab treatment was reported to increase the expression of p27^Kip1^, a cyclin-dependent protein kinase inhibitor (CKI), and arrest cells in G1 phase^26^. Previously, it was reported that S-phase kinase-associated protein 2 (Skp2), the ubiquitin ligase subunit that specifically targets the negative cell-cycle regulator p27^Kip1^ for degradation, is overexpressed in various cancers, including human HNSCC; its expression levels are inversely correlated to those of p27^Kip1^ in these cells^27^. Skp2 has been associated with ubiquitination-mediated degradation of the cyclin-dependent kinase (CDK) inhibitor p27^Kip1^ both in vitro and in vivo, and it positively regulates the G1/S transition^28^. Skp2 activation is controlled by the Akt–mTOR pathway^29^. Previous data showed that Akt controls its own expression level via Skp2 phosphorylation^30,31^. Suppression of mTOR was also observed during autophagy induction^32^. Since the role of p27^Kip1^ in contact-dependent cell growth inhibition has been well described, we hypothesized that the reduced cell motility that occurs after prolonged cetuximab treatment augments cell density and cell-to-cell contact, which inhibits tumor cell growth. To test this hypothesis, we used SAS cells constitutively expressing a fluorescent ubiquitination-based cell cycle indicator (Fucci). Following continuous cetuximab treatment, we assessed clonal growth, cell motility, and cell-cycle progression using fluorescent time-lapse imaging over the course of 10 days. The current findings might improve our understanding of tumor biology following cetuximab treatment and might aid in the development of improved therapeutic strategy for HNSCC.

## Results

### Prolonged cetuximab treatment inhibits cell growth in SAS cells in vitro

**Figure 1:**
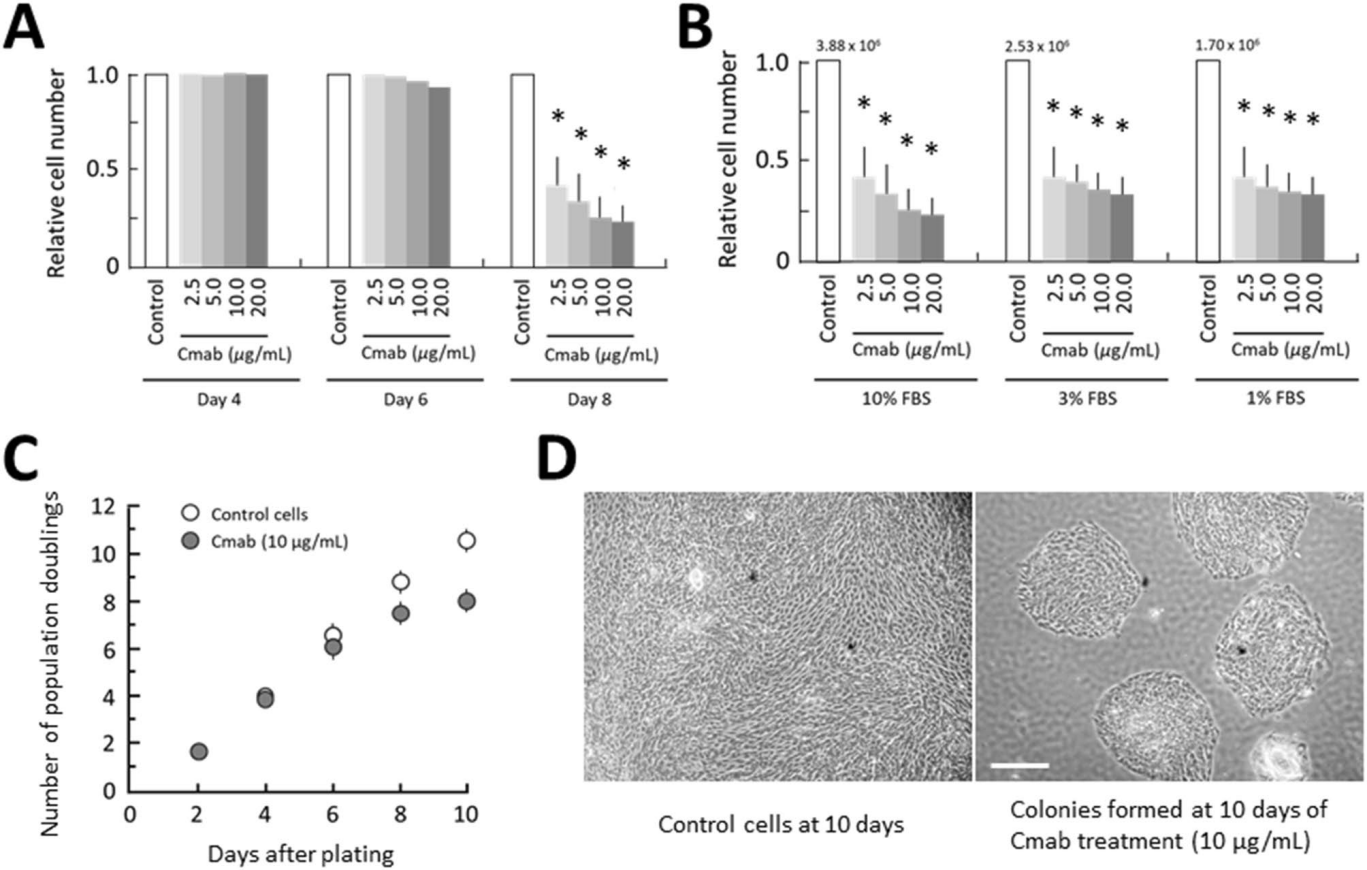
Delayed anti-tumor effect of cetuximab. **(A)** The anti-tumor effect of cetuximab (Cmab) was not apparent during the first few days of treatment, but dose-dependent growth inhibition was observed by the eighth day of treatment. *p < 0.001. **(B)** The serum concentration did not block the effect of the cetuximab antibody (10 µg/mL) on day 8. *p < 0.001. FBS, fetal bovine serum. **(C)** Total cells were counted, and the number of population doublings was calculated every 2 days. **(D)** The same numbers of cells were plated and cultured for 10 days. The control cells kept growing until they became confluent. In contrast, cells treated with cetuximab (10 µg/mL) showed delayed inhibition of cell growth, such that the arrested cells formed clonal colonies. Data are represented as means ± SD of three independent experiment. Scale bar, 500 µm.

Forty-eight hours after SAS-Fucci cells were plated, cetuximab was added to the culture medium at one of several different concentrations (2.5, 5, 10, or 20 µg/mL). The anti-tumor effects of cetuximab were determined by measuring cell-growth inhibition. As shown in Fig. 1A, no growth inhibition was observed during the first 4 days of treatment, although significant growth inhibition was found on day 8. Marked growth suppression was observed using cetuximab at a concentration of 10 µg/mL, and therefore that concentration was used in subsequent experiments. Unexpectedly, the growth-suppressive effect did not depend on the serum concentration at 8 days of treatment (Fig. 1B). Temporal cell-growth analysis confirmed that cell growth was not affected by cetuximab administration by 6 days. However, prolonged treatment (10 days) caused apparent growth suppression (Fig. 1C), which was exhibited by limited clonal outgrowth by the treated cells (Fig. 1D).

### Prolonged cetuximab treatment induces central cells in SAS colonies into G1 phase cell cycle arrest

**Figure 2:**
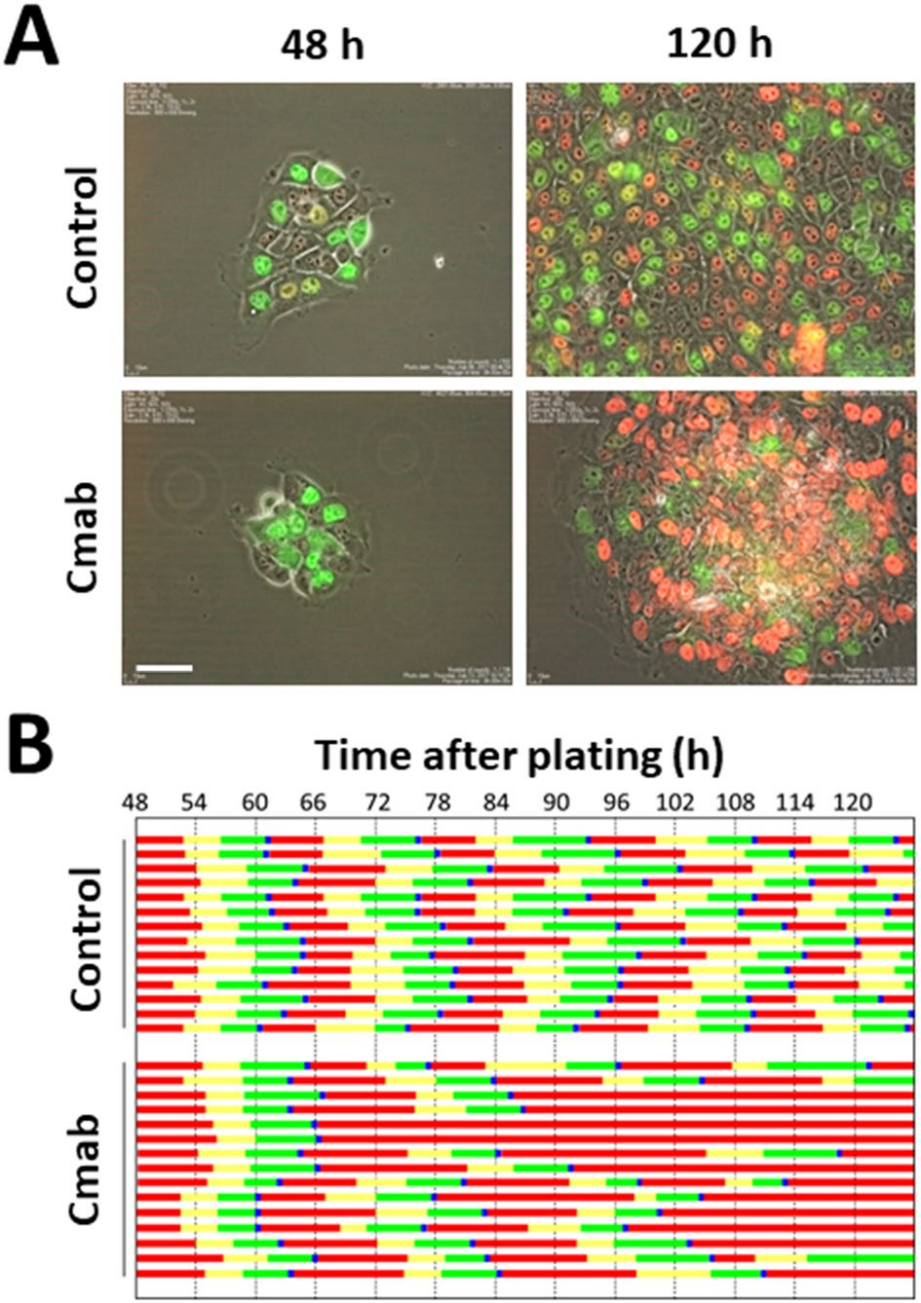
Time-lapse and pedigree analysis of control- and cetuximab-treated SAS-Fucci cells. **(A)** Cetuximab was administered to SAS-Fucci cells 48 h after they were plated in cell culture dishes. Time-lapse analysis using the Fucci system revealed that cells in G1 phase (red) gradually accumulated especially in the central regions of colonies after 120 h of treatment. In contrast, the control cells showed stable growth. **(B)** Pedigree assays revealed that, after treatment with cetuximab (10 µg/mL), the cells in the central regions of colonies gradually became arrested in G1 phase, whereas the control cells continued to divide once per ~ 20 h. Red, yellow, green, and blue bars represented G1, S, G2, and M phase, respectively. Scale bar, 50 µm.

The cell-cycle dynamics were observed by performing time-lapse analysis of SAS-Fucci cells, based on fluorescence imaging of live cells. Cells arrested in G1 phase were monitored for 5 days. While homogeneous cell growth and cell-cycle progression were observed in the control cells (Fig. 2), cetuximab treatment caused limited clonal outgrowth (Fig. 2A, 120 h), and more cells in G1 phase were evident in the central regions of the colonies. Our results showed that many of the cells in the central regions of the colonies were persistently arrested in G1 phase (Fig. 2B), whereas cells lining the colony rims showed stable growth, which was comparable to the control cells.

### Prolonged cetuximab treatment decreases motility of SAS cells in vitro

**Figure 3:**
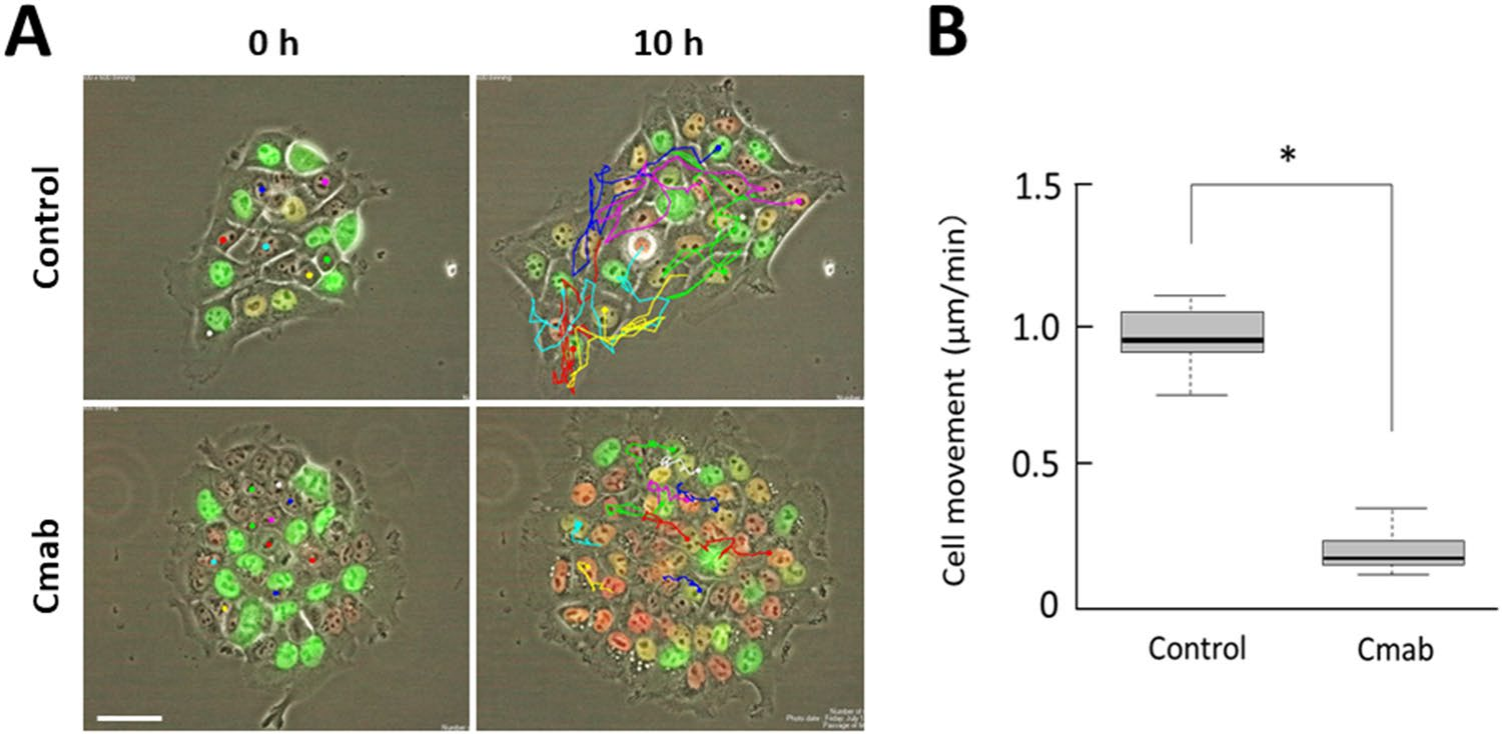
Movement velocities of control- and cetuximab-treated SAS-Fucci cells. **(A)** The movements of individual SAS-Fucci cells in small clusters were monitored for 10 h. **(B)** The velocity of cell movement, calculated using the TrackMate application, decreased significantly by the fifth day treatment with cetuximab (10 µg/mL). Data are represented as box-whisker plots showing outliers, distribution intervals, 25–75% interquartile range (box), and median. *p < 0.001. Scale bar, 50 µm.

Time-lapse imaging analysis was also applied to quantify cell motility. The movements of SAS-Fucci cells growing in small clusters were tracked for 10 h (Fig. 3A). The control cells clearly exhibited motility; however, the cetuximab-treated cells showed very limited motility. Calculating the velocity of each cell demonstrated that cetuximab treatment significantly diminished cell motility (*p* < 0.001) (Fig. 3B).

### Prolonged cetuximab treatment enhances mKO2-Cdt1 levels in growth-arrested SAS cells

**Figure 4:**
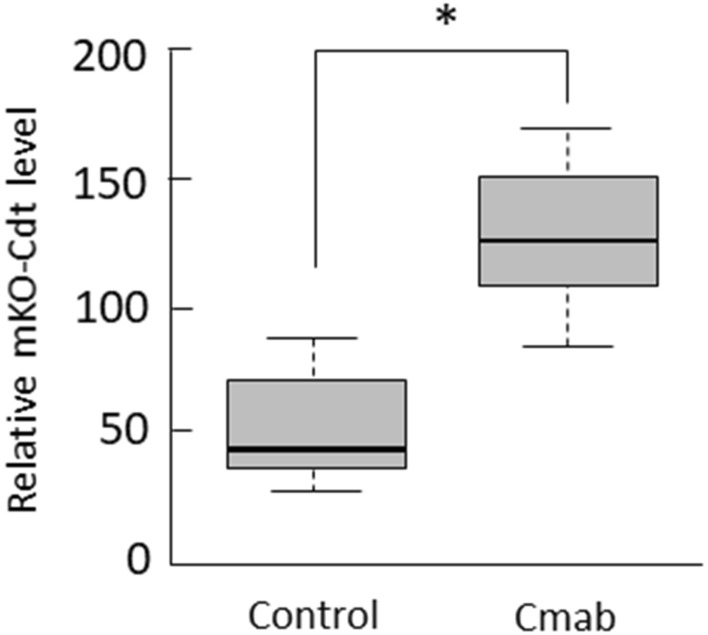
The mKO2-Cdt1 expression levels in fifth-day control- and cetuximab-treated SAS-Fucci cells. The mKO2-Cdt1 expression levels in individual G1 phase-arrested cell were significantly higher after treatment with cetuximab (10 µg/mL) when compared to control cells. Data are represented as box-whisker plots showing outliers, distribution intervals, 25–75% interquartile range (box), and median. *p < 0.05.

Live-imaging analysis indicated that cetuximab-mediated G1 arrest was mainly detected in the cells occupying the central regions of the colonies. Since high G1 red expression was directly derived from high mKO2-Cdt1 expression, our results indicated that cetuximab treatment may have caused abnormal expression of proteins involved in cell growth. Mann–Whitney U test revealed that the mKO2-expression levels were significantly higher in each G1-arrested cell following cetuximab treatment than in each G1 control cell (Fig. 4).

### Prolonged cetuximab treatment decreases the expression of ERK1/2, p38, p21, RB, p-RB, and p-Skp2, and enhances p27^Kip1^ levels

**Figure 5:**
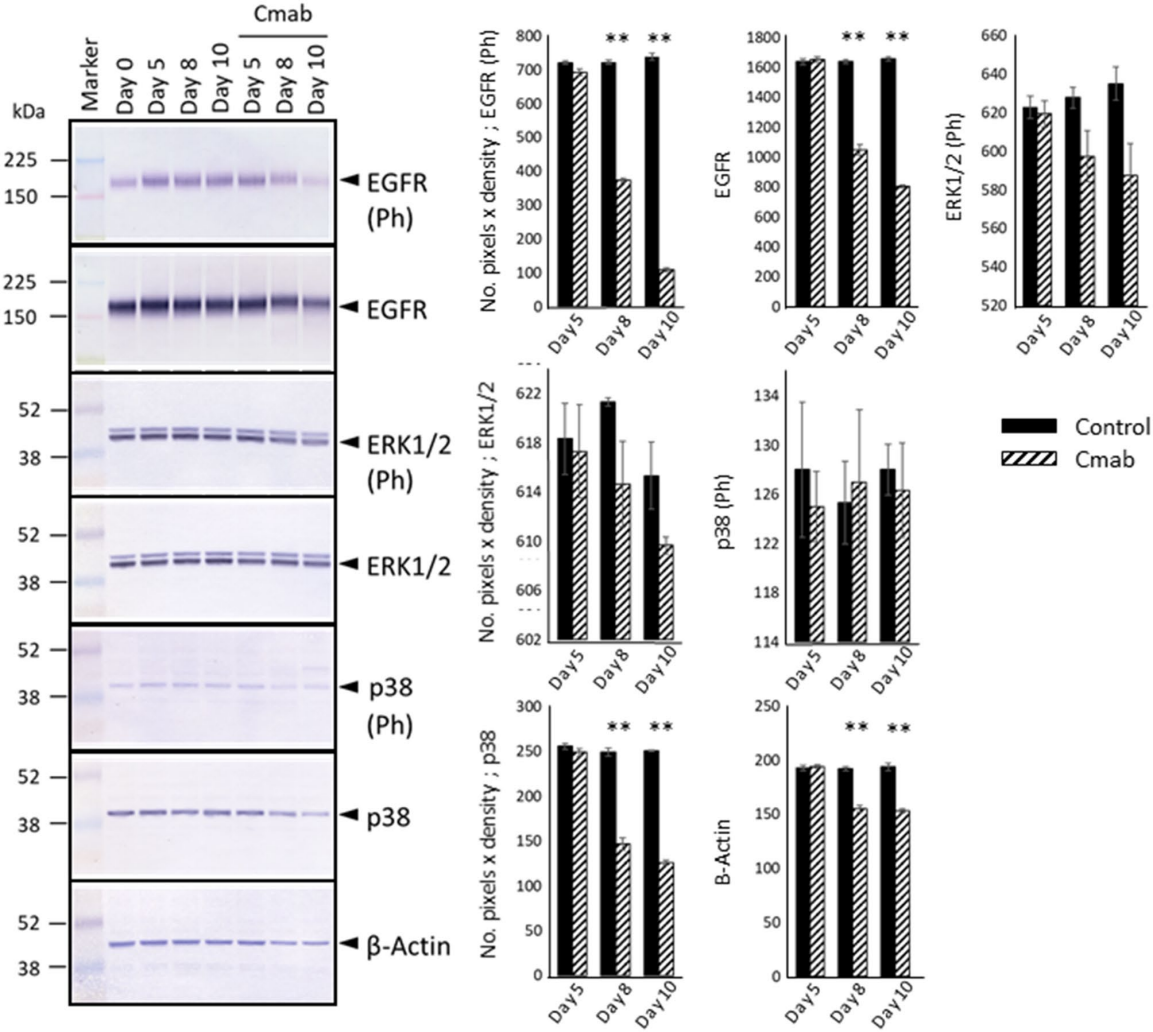
Western blotting (WB) analysis of control- and cetuximab-treated SAS cells. WB revealed the phosphorylation levels of ERK1/2, which are regulated by the EGFR pathway, decreased slightly after 8 days of cetuximab treatment. There was also no evidence of p38 activation, based on the levels of phosphorylated p38. Those expression levels were evaluated with the number of pixels × density and analyzed with t test (control- versus cetuximab-treated group). The experiment was repeated 3 times. *p < 0.05, **p < 0.001.

**Figure 6:**
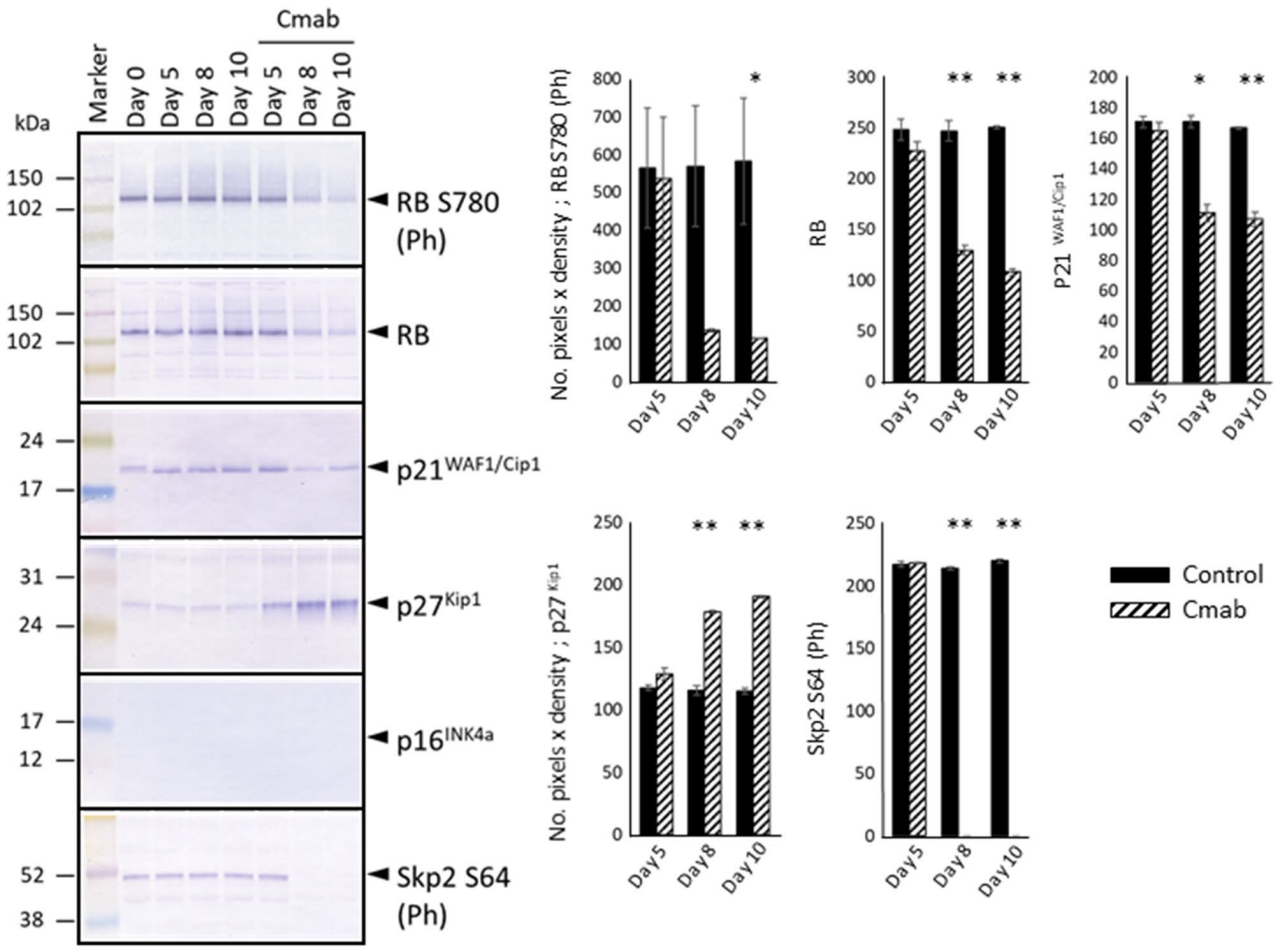
RB and phosphorylated-RB protein levels decreased considerably after 8 or 10 days of cetuximab treatment. Thus, the expression levels of other CKIs were investigated. The p21 protein-expression level decreased as well. The p16 protein was not detected in control- or cetuximab-treated SAS-Fucci cells. Instead, strong p27^Kip1^ expression was detected in SAS-Fucci cells treated with cetuximab (10 µg/mL) for 5 days. This examination revealed that prolonged cetuximab treatment inactivated the phosphorylation and function of Skp2. Those expression levels were evaluated with the number of pixels × density and analyzed with t test (control- versus cetuximab-treated group). The experiment was repeated 3 times. *p < 0.05, **p < 0.001.

**Figure 7:**
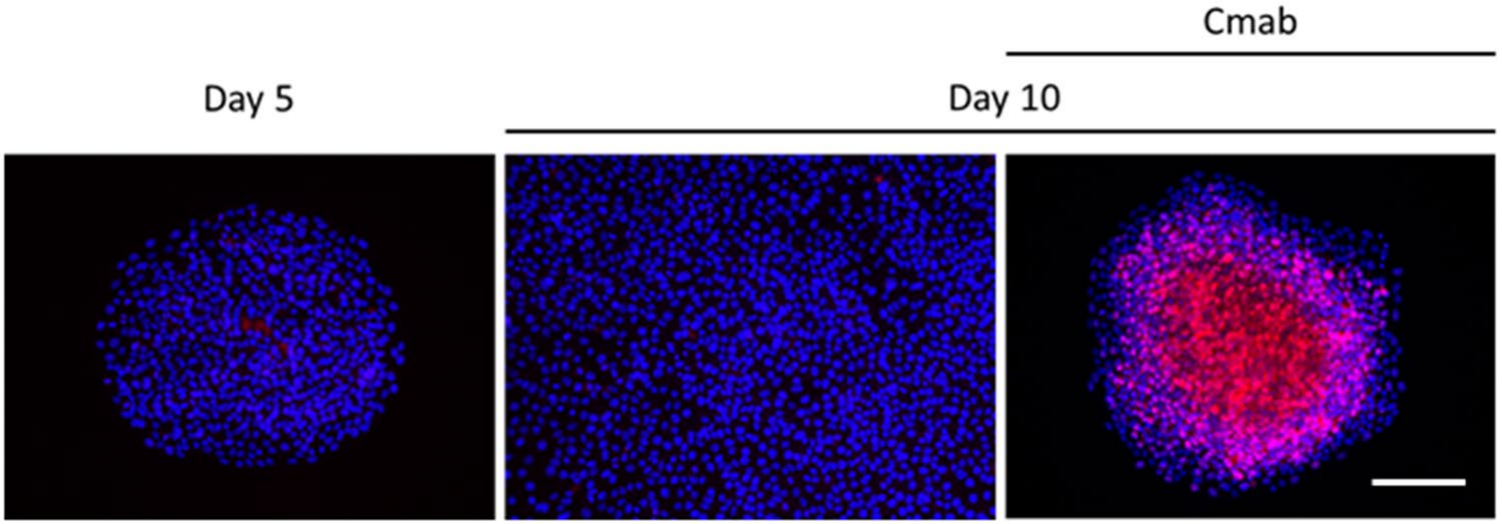
Immunofluorescent staining also confirmed that p27^Kip1^ was highly expressed in the central regions of colonies that formed after treatment with cetuximab (10 µg/mL) for 10 days, which was not observed in control cells following a 10-day treatment. This expression was not also detected in the same sized colonies which were established with 5-day incubation. Scale bar: 500 µm.

The effects of prolonged cetuximab treatment on cell growth-associated signaling pathways were examined by WB analysis. The phosphorylation levels of ERK1/2 did not change during the first 5 days of treatment and slightly decreased thereafter. Similar results were obtained in terms of p38 (Fig. 5, Supplementary Information). In contrast, both the total-RB and phosphorylated-RB protein levels decreased considerably after 8 or 10 days of cetuximab treatment. To study these expression differences mechanistically, we further investigated differences in the expression levels of CKIs. We found that p21 protein expression was not induced, but in fact decreased after 8 or 10 days of cetuximab treatment. The p16 protein was not detected in the SAS cells. The expression of p27^Kip1^, another CKI involved in contact inhibition, was clearly increased after 8 or 10 days of cetuximab treatment (Fig. 6, Supplementary Information). In addition, elevated p27^Kip1^ levels were predominantly observed in the central regions of the colonies (*p* < 0.001) (Fig. 7). p27^Kip1^ expression is regulated by Skp2, whose activity is regulated by phosphorylation^33^. Importantly, Skp2 phosphorylation was markedly compromised by cetuximab treatment (Fig. 6).

### Prolonged cetuximab treatment inhibits Skp2 and induces autophagy

**Figure 8:**
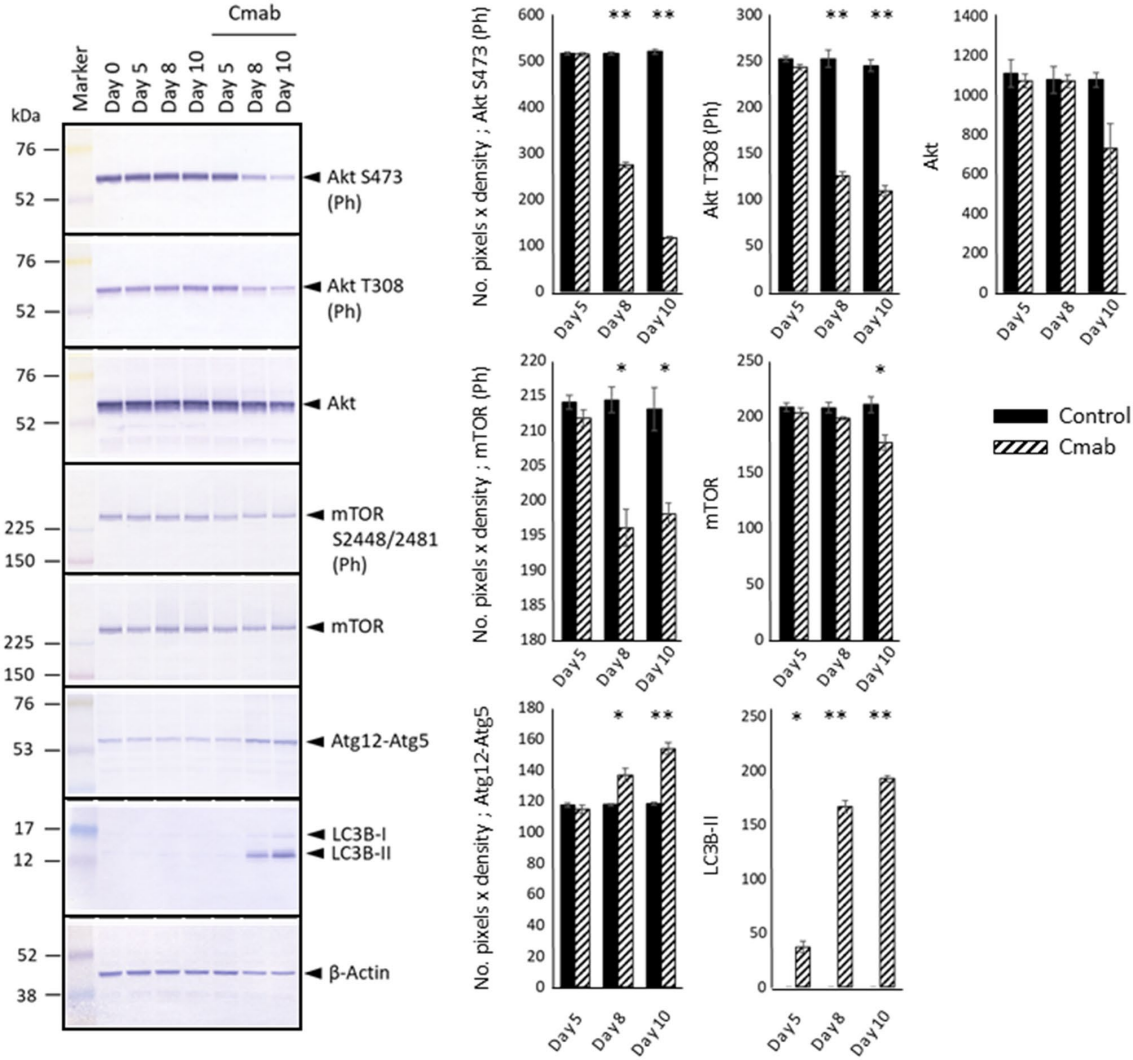
Examination of the Akt–mTOR pathway, which controls Skp2 activation. WB analysis revealed that cetuximab treatment (10 µg/mL) potently inhibited Akt phosphorylation at S473 and T308, which are related to Akt activation. In addition, cetuximab treatment suppressed mTOR phosphorylation at S2448 and S2481, which are related to mTOR activation. The expression levels of Atg12–Atg5 and LC3Bs, which are associated with autophagy, were also analyzed, which revealed markedly increased expression of the Atg12–Atg5 complex and type-II LC3B in cells treated with cetuximab for 8 or 10 days. Those expression levels were evaluated with the number of pixels × density and analyzed with t test (control- versus cetuximab-treated group). The experiment was repeated 3 times. *p < 0.05, **p < 0.001.

Because the phosphorylation and activity of Skp2 is controlled by Akt and mTOR^28–30^, we next analyzed Akt phosphorylation at S473 and T308, as well as mTOR phosphorylation at S2448 and S2481. We found that prolonged cetuximab treatment suppressed the phosphorylation of both proteins (Fig. 8, Supplementary Information). The observation of down-regulated mTOR phosphorylation was indicative of autophagy induction; therefore, we examined the expression levels of Atg12–Atg5 and LC3Bs. As shown in Fig. 6, the levels of the Atg12–Atg5 complex and type-II LC3B notably increased in cells treated with cetuximab for 8 or more days.

## Discussion

Cetuximab has been described as a promising monoclonal antibody that can inhibit the growth of HNSCCs. While p27^Kip1^-dependent cell-cycle arrest was involved in the anti-tumor effect, its induction mechanism remains to be determined. In this study, we discovered that the growth-inhibitory effect of cetuximab became apparent only after continuous treatment for more than 5 days, indicating that cell-cycle arrest was not induced by the immediate cellular responses initiated by EGFR inhibition. Therefore, the temporal cell-cycle dynamics were examined using the Fucci system, which is a fluorescent ubiquitination-based cell-cycle indicator that causes cells to emit red fluorescence in G1 phase and green fluorescence in S/G2/M phases^34^. Pedigree assays confirmed that cell-cycle progression was similar between the control and cetuximab-treated cells by 3 days; however, treated cells (particularly those in the central regions of forming colonies) eventually became arrested at G1 phase. We also found that cell motility was significantly diminished by continuous cetuximab treatment, indicating that a mutual relationship may exist between reduced cell motility and cell-growth suppression.

Protein analyses were performed to evaluate the inhibitory effects of cetuximab on EGFR-dependent signaling pathways. Although several previous reports have demonstrated that cetuximab treatment can immediately suppress EGFR phosphorylation and downstream signaling pathways^8–12^, few studies have been performed to investigate the effects of prolonged treatment. Our results demonstrated that ERK1/2 phosphorylation, which is predominantly regulated by EGFR signaling, was not affected by cetuximab treatment  in SAS-Fucci cells. Oncogenic mutation profiles of SAS cell line have been reported in several previous studies, demonstrating mutations in the p53 and NOTCH1 genes but not in the H-ras, BRAF, MEK1/2 and ERK1/2 genes^35–37^. Mutation in the HER4 and CNV gain of the K-ras gene were also reported^38^. These mutations could explain why the minimal suppressive effect of cetuximab on EGFR phosphorylation was observed in the present examination.

It is well recognized that continuous treatment with a MAP kinase inhibitor causes a phenomenon known as adaptation, wherein cells acquire resistance to the MAP kinase inhibitor due to the elimination of negative feedback^39^. EGFR inhibition has also been associated with acquired resistance^40,41^. Therefore, the delayed inhibition of cell growth observed in this study must have been caused by another mechanism, which could be cell-to-cell contact stress stemming from reduced cell motility. Indeed, our observations demonstrated that reduced cell motility correlated with a higher cell density. For example, cells in the central regions of growing colonies showed restricted movement and arrest in G1 phase. In agreement with a previous report^26^, we also observed that p27^Kip1^ was elevated after prolonged treatment with cetuximab. Moreover, we demonstrated that this phenomenon was closely related to a high cell density.

In this study, we utilized live-cell imaging and time-lapse analysis, which revealed that cetuximab treatment significantly reduced cell motility. Previous investigators reached the same conclusion after performing cell-migration assays^25,42,43^. Thus, we conclude that reduced cell motility was associated with a high cell density. The mechanism that led to the reduction in cell motility remains unclear. Although the associated mechanism was not identified in this study, several related observations have suggested that EGFR inhibition is interrelated with reduced cell migration. For example, it was reported that cetuximab inhibited the epithelial–mesenchymal transition of HNSCC, indicating that strengthening cell-to-cell contacts limited cell motility^24^. Increased actin filaments were also observed after cetuximab treatment^42^. Thus, future studies should be performed to identify the underlying mechanism by which cetuximab treatment alters cell adhesion and the organization of the cytoskeleton. The fate of those cells was also unclear; G1-arrested SAS-Fucci cells, then, was not apparently observed apoptosis but irreversible senescence-like arrest was observed in most cells. Thus, we believe this phenomenon induces senescence-like cell death. This senescence and mechanism to subsequent cell death also should be investigated in the future.

**Figure 9:**
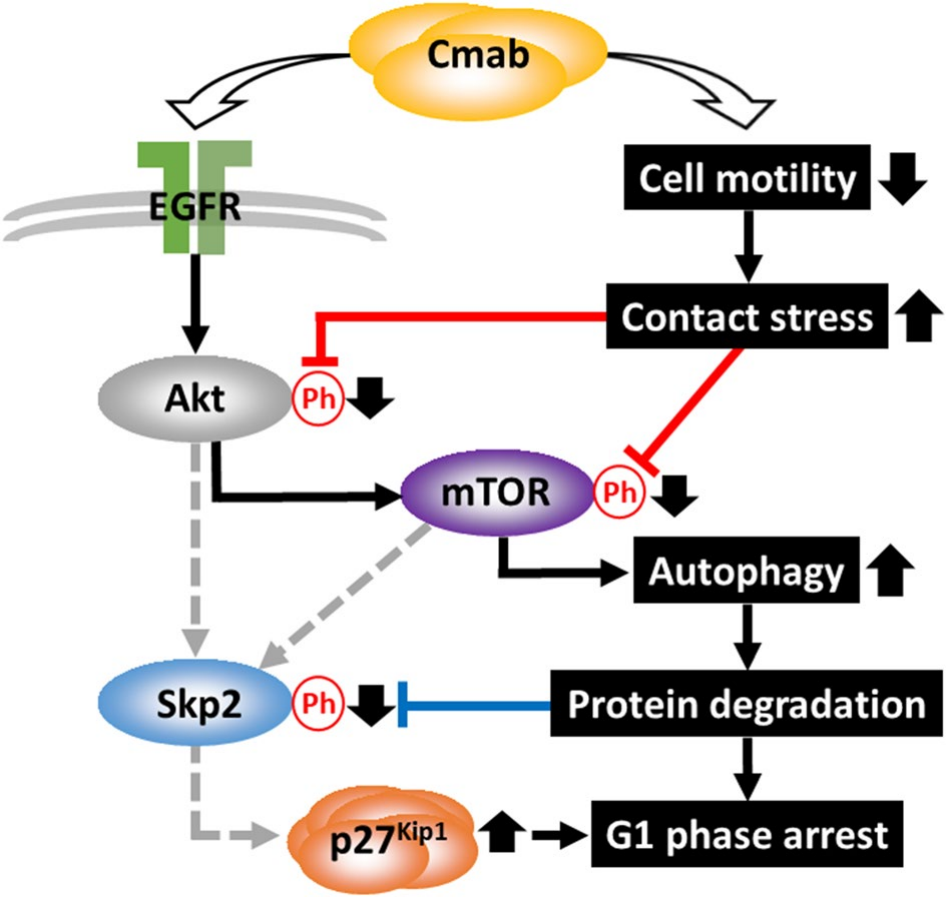
Mechanism of p27^Kip1^ accumulation and autophagy induction following cetuximab treatment. Continuous inhibition of cell migration induced by prolonged cetuximab treatment resulted in decreased Akt and mTOR phosphorylation (red lines), which subsequently decreased Skp2 phosphorylation (blue line) and led to p27^Kip1^ accumulation, due to the suppressed degradation of p27^Kip1^. The down-regulation of mTOR phosphorylation was also associated with autophagy induction.

Considering that decreased phosphorylation of Akt^25^ and mTOR^44^ was previously observed in high-density cells, our current results suggest that reduced cell motility indeed played a critical role in inducing contact stress, which was reported to suppress Akt and mTOR phosphorylation^45^. Because the Akt and mTOR kinases are indispensable for Skp2 phosphorylation, we examined Skp2 phosphorylation during prolonged treatment with cetuximab. Our results clearly indicated that Skp2 phosphorylation was significantly abrogated by continuous cetuximab treatment; conversely, the expression of p27^Kip1^ (which is degraded by Skp2) was increased. In addition, decreased mTOR phosphorylation led to mTOR down-regulation, which resulted in autophagy induction, as demonstrated by changes in the expression of autophagy-related proteins. These findings raise the possibility that treatment with an anti-EGFR antibody might be effective, even in cases where cancer cells have acquired resistance to inhibition of the EGFR-signaling pathway (Fig. 9). Previously, K-Ras mutations in colon cancer were clinically associated with a lower efficacy of cetuximab^46,47^. However, p27^Kip1^ accumulation and autophagy induction may delay the anti-tumor effects of cetuximab independently of whether such mutation are present. Furthermore, abnormal protein-expression levels of Skp2 and p27^Kip1^ have been detected in many malignancies; both proteins play important roles in the pathogenesis and development of malignant tumors, and their expression levels also impact patient prognosis^28,48,49^. In future studies, the effects of cetuximab should be comprehensively examined in tumors with different combination of oncogenic mutations, in light of the findings presented above. In addition, combined treatment with cetuximab and targeted therapeutics against CDKs may exhibit synergistic anti-tumor effects, and both in vivo and in vitro studies should be performed in the future. Considering that radiation therapy can induce autophagy^50^, cetuximab treatment by itself is expected to serve as a radiosensitizer. On the other hand, as Wang et al. previously reported that cetuximab-mediated autophagy conversely induced radioresistance on cancer cells; autophagy itself could also demonstrate radioresistance^51^. The clinical meaning of autophagy should be examined. Moreover, since cetuximab shows high antibody-dependent cell-mediated cytotoxicity^12,21,22^ and radiotherapy can potentiate innate immunity by activating the cGAS-STING pathway^52^, the immunobiological effects of cetuximab and combined therapy with radiotherapy should be also examined carefully.

In conclusion, cetuximab treatment in SAS cells did not demonstrate early inhibition of the EGFR pathway; however, prolonged treatment resulted in p27^Kip1^ accumulation and autophagy induction via down-regulation of Akt and mTOR phosphorylation, which was caused by cell-contact stress and decreased cell motility.

## Methods

### Cell lines and culture conditions

A human tongue SCC cell line, SAS, which was transfected Fucci system (SAS-Fucci cell) as described previously was used in this study^53^. SAS-Fucci cells were maintained in DMEM (Sigma-Aldrich, St. Louis, MO, USA) containing a high concentration of glucose (4500 mg/L) with 100 units/mL penicillin and 100 µg/mL streptomycin, which was supplemented with 10% fetal bovine serum (FBS), at 37 °C in a humidified 5% CO_2_ atmosphere. We also performed experiments with several concentrations of FBS (1, 3, and 10%) to confirm that EGF (a component of FBS) would not affect EGFR activity and cell proliferation. Cells were plated at a sparse density (10^3^ cells/dish) in 35-mm culture dishes, so that individual colonies could form.

### Drug preparation and proliferation assay

Cetuximab (C225, Erbitux™) was obtained from Merck (Darmstadt, Germany). Working solutions were freshly prepared from a stock solution (5 mg/ml) by dilution in DMEM (Sigma-Aldrich, St. Louis, MO, USA) containing a high concentration of glucose (4500 mg/L) with 100 units/mL penicillin and 100 µg/mL streptomycin, which was supplemented with 10% FBS, on the day of the experiment. The working solution was replaced every 2 days during incubation. SAS-Fucci cells were plated in 35-mm culture dishes at a density of 5 × 10^3^ cells/dish and expanded for 48 h, after which they were treated with cetuximab. Subsequently, the cells were collected at different time points using trypsin and counted with an automated cell counter (TC20, Bio-Rad laboratories, Tokyo). Population doubling was calculated based on the number of cells at the end point.

### Live-cell imaging

Time-lapse phase-contrast and fluorescent images of cells were obtained using a BioStation ID microscope (GE Healthcare Bioscience, Tokyo). Cells were plated onto a glass-bottomed 35-mm dish at a density of 5 × 10^4^ cells/dish and incubated for 24 h before the cetuximab treatment. Working solution with cetuximab was replaced at first 48 h from the beginning of observation. Fifth days cetuximab- and control-treated SAS cells were imaged. During imaging, the cells were maintained at 37 °C in a humidified atmosphere containing 95% air and 5% CO_2_. For quantitative analysis, fluorescent intensities were measured using multiple randomly selected areas to generate the optical-density plots using the Histogram tool in the Image menu of Adobe Photoshop (Adobe Systems, Inc., San Jose, CA, USA) in cell clusters after fifth days cetuximab treatment. The average fluorescent intensities within the randomly selected images were calculated.

### Cell-motility assay

Cell motility was analyzed using image-based software obtained from the Fiji-ImageJ plugin "TrackMate" (Tinevez)^54^. Briefly, live-cell images of fifth days cetuximab- and control-treated SAS cells were acquired every 10 min using a BioStation ID microscope (GE Healthcare Bioscience, Tokyo) as described above, and the movements of the assigned cells were tracked for over 10 h. Then, the total movements were averaged, and the average velocities of cell movement were calculated according to the TrackMate protocols.

### Western blot (WB) analysis

The working solution was replaced every 2 days during incubation. Following 5, 8, or 10 days of continuous cetuximab treatment, the treated and control samples were collected on same day, and proteins were extracted for WB analysis. Total cell extracts were prepared using Qproteome mammalian protein prep kit (Qiagen Japan, Tokyo). The cell lysate was cleared by centrifuging it at 15,000 rpm for 10 min at 4 °C. Then, the supernatant was collected and used as the total cellular protein fraction. Protein concentration was determined using the bicinchoninic acid protein assay (Pierce, Rockford, Ill.). Total proteins (8 µg) were electrophoresed through sodium dodecyl sulfate polyacrylamide gel and transferred electrophoretically to a polyvinyl difluoride membrane in transfer buffer (100 mM Tris and 192 mM glycine). After 30 min’ incubation with blocking solution (10% skimmed milk in TBS-T buffer, 20 mM Tris–HCl, pH 7.6, 137 mM NaCl, 0.1% Tween 20), the membrane was incubated with the primary antibodies, biotinylated anti-mouse or rabbit immunoglobulin G antibodies, and streptavidin–alkaline phosphatase. To visualize the resultant bands, the membrane was incubated in a detection solution containing nitroblue tetrazolium/5-bromo-4-chloro-3-indolyl phosphate (Merck Japan, Tokyo) as a substrate. Each blot was scanned by a high-resolution scanner, and the relative density of each band was quantified using ImageJ software. Then, the expression level of each protein was normalized to the expression level of β-actin, and the relative expression levels of each protein compared with those seen on day 0 were calculated. Antibodies against the following proteins were obtained from Cell Signaling Technology Japan (Tokyo) for use as primary antibodies: EGFR (#4267), EGFR phosphorylated at Y1068 (#2234), ERK1/2 (#4695), phosphorylated ERK1/2 (#4370), p38 (#9212), phosphorylated p38 (#4511), RB phosphorylated at S780 (#8180), RB (#9313), Skp2 phosphorylated at S64 (#14,865), Akt (#4691), Akt phosphorylated at S473 and T308 (#2965 and #4060, respectively), mTOR phosphorylated at S2448 and S2481 (#2974 and #5536, respectively), mTOR (#2983), Atg12-Atg5 (#2010), and LC3B (#12,741). Primary antibodies against p21^WAF1/Cip1^ (ab107099), p27^Kip1^ (ab32034), and p16^INK4a^ (ab117443) were obtained from Abcam, PLC (Cambridge, UK). A primary antibody against β-Actin (2F1-1) was obtained from BioLegend (San Diego, USA). The biotinylated anti-mouse or rabbit IgG antibodies and streptavidin–alkaline phosphatase were obtained from Amersham (Amersham Japan, Tokyo).

### Immunofluorescence

Cells were seeded on glass coverslips (22 × 22 mm, Matsunami, Tokyo, Japan) placed in 35-mm dishes. After 24 h incubation, cetuximab (10 µg/ml) treatment was performed for 10 days. All samples were fixed in 4% formaldehyde for 10 min, permeabilized with 0.1% Triton X-100 in PBS for 5 min, and washed extensively with PBS. The cells were incubated at 37 ºC for 2 h with the following primary antibodies: rabbit anti-p27^Kip1^ (ab32034, Abcam PLC) to evaluate accumulation of the p27 protein. In addition, DAPI (1 µg/ml, Sigma-Aldrich, St. Louis, MO, USA) was used for nuclear staining. Slides were then incubated for 1 h with an Alexa Fluor 555-conjugated anti-rabbit IgG secondary antibody (A32732, Life Technologies, CA, USA). The slides were mounted using PBS containing 10% glycerol and sealed with glass coverslips.

Images were captured with a DFC-350 FX digital camera (Leica, Germany) attached to the fluorescent microscope, and randomly selected areas were analyzed to generate optical-density plots using the Histogram tool in the Image menu of Adobe Photoshop (Adobe Systems, Inc.). The average fluorescent intensities within the selected areas were recorded as arbitrary units, and the relative fluorescent intensities protein (monomeric Kusabira-Orange 2 (mKO2)) were calculated to evaluate the frequency of G1-arrest in fifth-day cetuximab treatment cells.

### Statistical analysis

Experiments were repeated at least three times. All statistical analyses were conducted using SPSS software for Windows Version 25.0 (Armonk, NY: IBM Corp). Cell-growth rates were assessed by one-way ANOVA. Cell motilities and mKO2-expression levels were analyzed using the Mann–Whitney U test. The protein expression levels, which were calculated by the number of pixels × density on WB analysis, were expressed as mean ± standard error; differences between the control- and the cetuximab-treated group were tested by t test. Two-sided *P-*values of < 0.05 were considered to reflect statistically significant differences.

### Ethical approve

This research need not to obtain the approval of IRB in the institute, because this basic research was performed using only cell line and did not have any patient’s information.

## Supplementary Information

Supplementary Figures.
