# Supplementary material for: Prolonged cetuximab treatment promotes p27Kip1-mediated G1 arrest and autophagy in head and neck squamous cell carcinoma
Source: Sci Rep. 2021 Mar 4;11:5259. doi: 10.1038/s41598-021-84877-4 (PMC7933308; doi:10.1038/s41598-021-84877-4)

**Scientific Reports**

**[ Supplementary Information ]**

**Prolonged cetuximab treatment promotes p27^Kip1^-mediated G1 arrest and autophagy in head and neck squamous cell carcinoma**

Kohei Okuyama^1,2,3^, Keiji Suzuki^2^, Tomofumi Naruse^3^, Hiroki Tsuchihashi^3,4^, Souichi Yanamoto^3^, Atsushi Kaida^5,6^, Masahiko Miura^6^, Masahiro Umeda^3,^ Shunichi Yamashita^2,7,8^

1 Department of Oral and Maxillofacial Surgery, Graduate School of Medical and Dental Sciences, Tokyo Medical and Dental University, Tokyo, Japan.

2 Department of Radiation Medical Sciences, Atomic Bomb Disease Institute, Nagasaki University, Nagasaki, Japan.

3 Department of Clinical Oral Oncology, Nagasaki University Graduate School of Biomedical Sciences, Nagasaki University, Nagasaki, Japan.

4 Department of Maxillofacial Diagnostic and Surgical Science, Field of Oral and Maxillofacial Rehabilitation, Graduate school of Medical and Dental Sciences, Kagoshima University, Kagoshima, Japan.

5 Department of Cancer Biology, The University of Kansas Medical Center, Kansas City, KS, USA.

6 Department of Oral Radiation Oncology, Division of Oral Health Science, Graduate School of Medical and Dental Sciences, Tokyo Medical and Dental University, Tokyo, Japan.

7 Center for Global Exchange, Fukushima Medical University, Fukushima, Japan.

8 Center for Advanced Radiation Emergency Medicine, National Institutes for Quantum and Radiological Science and Technology, Chiba, Japan.

**Corresponding author:**

**Kohei Okuyama, DDS, PhD**

Research Assistant Professor, Department of Oral and Maxillofacial Surgery, Graduate School of Medical and Dental Sciences, Tokyo Medical and Dental University, Tokyo, Japan.

1-5-45, Yushima, Bunkyo-ku, Tokyo, 113-8510

Tel: +81-3-5803-5506

Email: [okuyamak.0429@gmail.com](mailto:okuyamak.0429@gmail.com)

**Key words:**

Akt-mTOR, type II LC3B, Skp2, cell motility, and clonal growth.

**Running head:** Anticancer effect of Prolonged cetuximab treatment

**Conflict of interest statement:**

The authors have no conflict of interest.


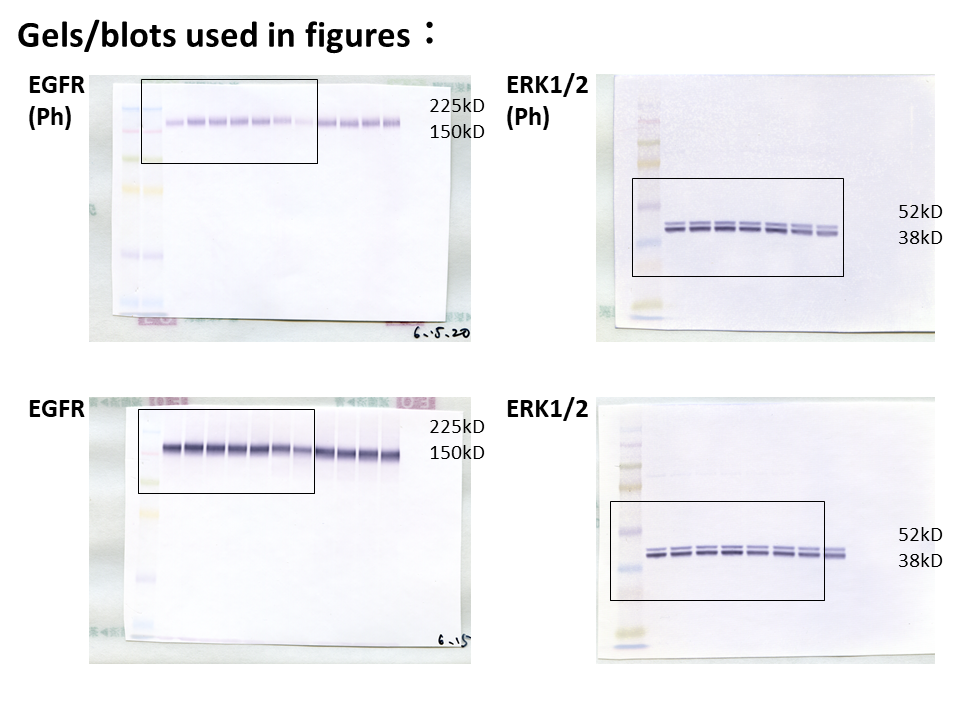


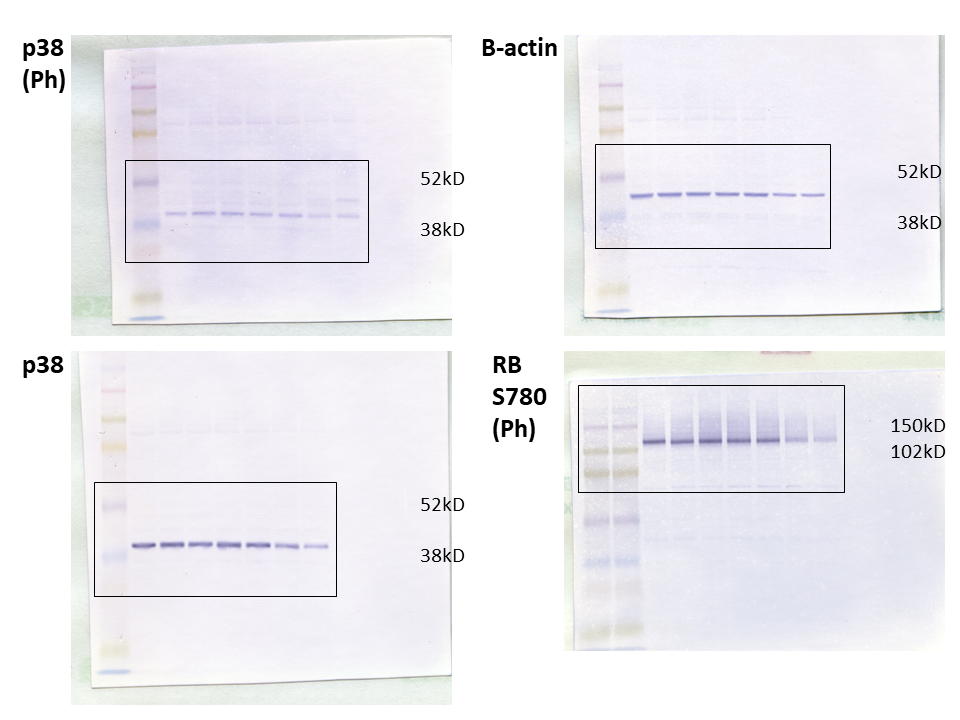


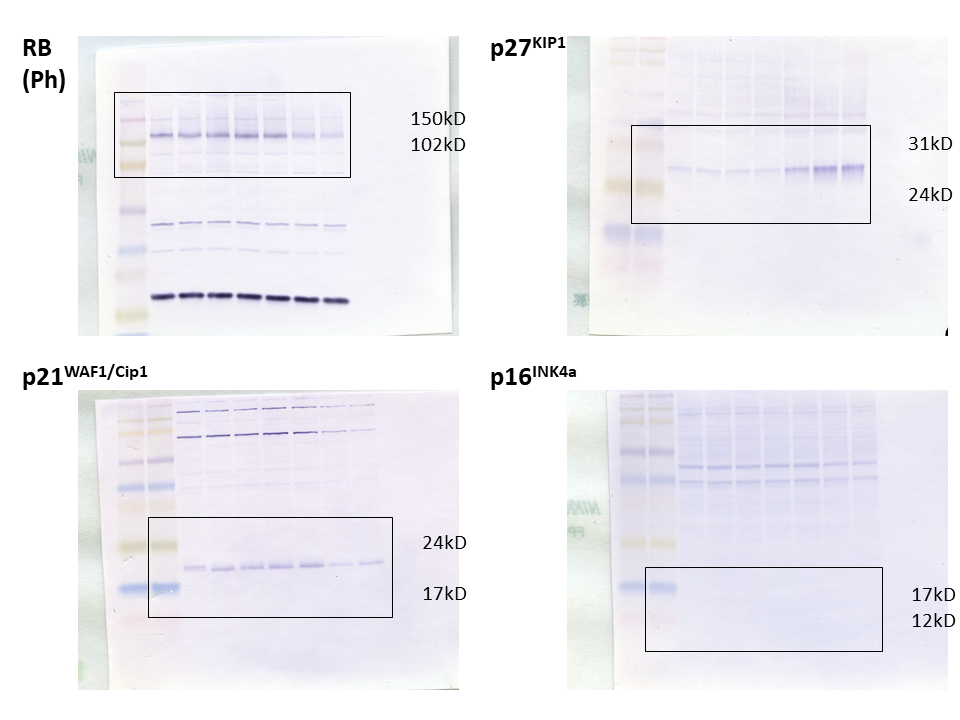


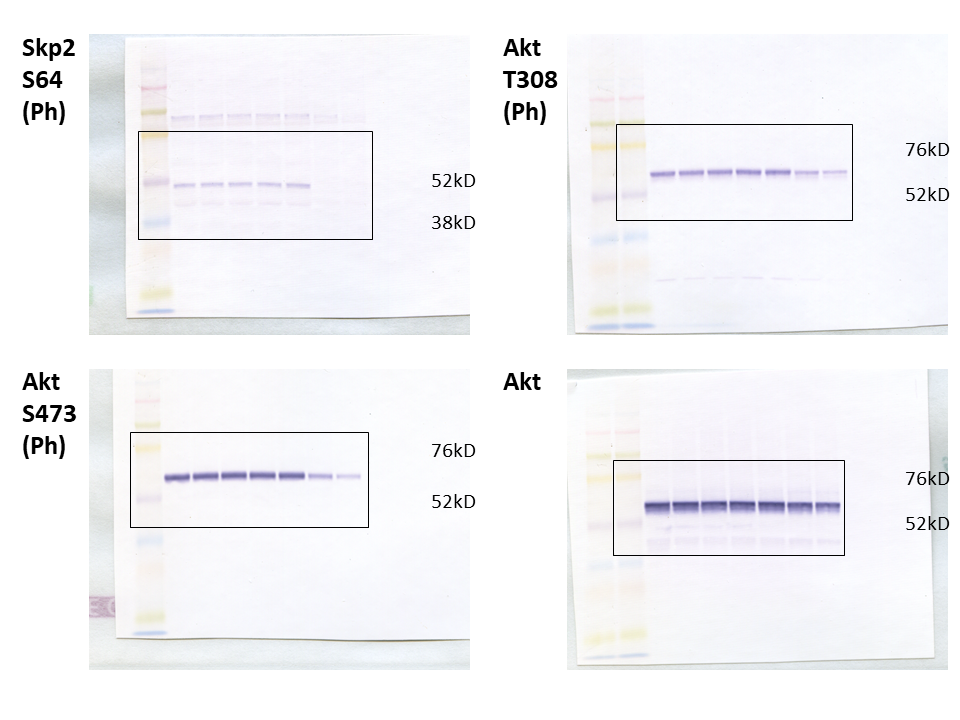


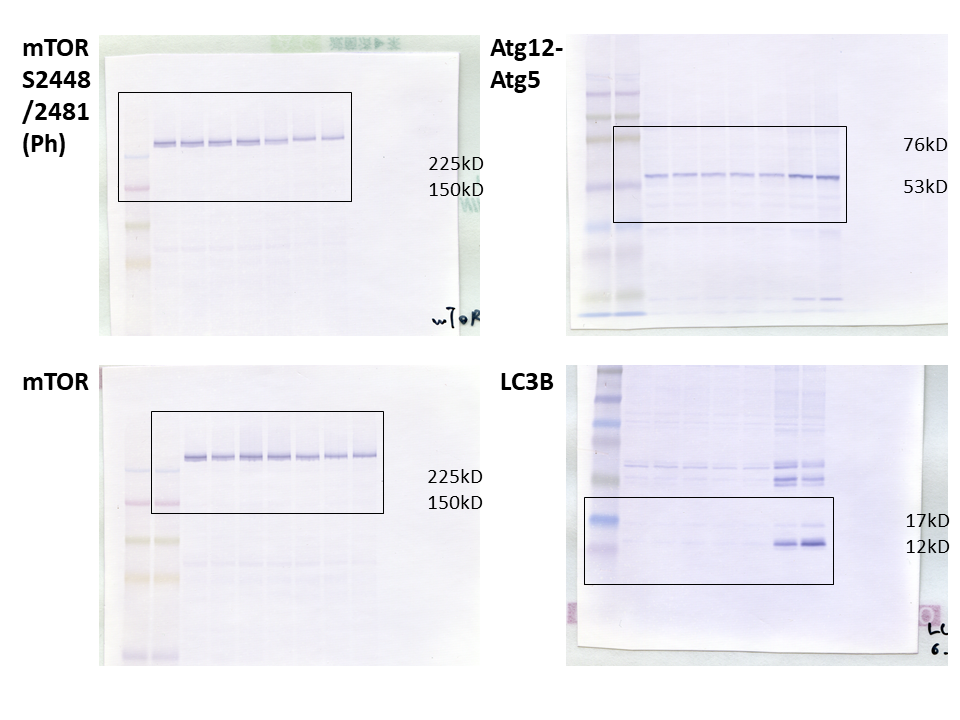

Supplement: Supplementary file 1 — Supplementary Figures. [file 41598_2021_84877_MOESM1_ESM.docx]
